# A concurrent process for the automatic preparation of biological samples combined with high pressure liquid chromatographic analysis

**DOI:** 10.1155/S1463924686000299

**Published:** 1986

**Authors:** D. C. Turnell, J. D. H. Cooper

**Affiliations:** The Department of Biochemistry Coventry & Warwickshire Hospital Stoney Stanton Road Coventry CV1 4FH UK


Journal of Automatic Chemistry/*Journal of Clinical Laboratory Automation*, Vol. 8, No. 3 (July–September 1986), pp. 151–154

### A concurrent process for the automatic preparation of biological samples combined with high pressure liquid chromatographic analysis

**D. C. Turnell and J. D. H. Cooper**

*The Department of Biochemistry, Coventry & Warwickshire Hospital, Stoney Stanton Road, Coventry CV1 4FH, UK*

### Introduction

The objectives of sample preparation for high pressure liquid chromatography (HPLC) analysis are the elimination of compounds that would otherwise progressively reduce the performance of the analytical column and directly interfere with the separation and quantitation of analytes. Approaches to achieve these objectives have included liquid–liquid extraction [1 and 2], protein precipitation [3], ultrafiltration [4], dialysis [5] and the use of sorbants, by either pre-column [6] or column switching and back-flushing techniques [7]. All these operate as batch processes with the exception of column switching methods and those employing robots.

The technique of automatic sequential trace enrichment of dialysates (ASTED) has been shown to be an inexpensive and effective technique for deproteinizing serum samples prior to HPLC analysis: the trace enrichment cartridge can be regenerated for subsequent analyses hundred of times [8 and 9]. However, it operated in a purely sequential mode with the HPLC, such that the second sample was not processed until the first had been analysed and consequently half of the system was always idle. This paper describes modifications to the basic ASTED system that enable concurrent processing of adjacent samples so that the second sample may be prepared whilst the first is being chromatographed.

### Equipment and processes

### *HPLC unit*

The HPLC equipment was loaned by Gilson International, Villiers-Le-Bel, France, and comprised two 303/5SC HPLC pumps, a 620 Data Master and 502 Contact Module, all controlled by a 704 System Manager (version 2.1) operating in an Apple IIe microcomputer.

### *Sample preparation unit*

The sample preparation unit comprised a modified Auto-Analyser sampler II and dialyser block fitted with a Cuprophan C-type membrane (molecular weight cut-off 10000 Daltons, Technicon, Basingstoke, Hampshire, UK), a Minipuls and 303/5SC HPLC pump (Gilson International, Villiers-Le-Bel, France), a Rheodyne 7010 injection valve with a 12 V pneumatic controller (Anachem, Luton, Bedfordshire, UK), a Sinclair Spectrum microcomputer and a purpose-built interface based on an Z80A parallel input/output controller (Radio Spares, London). The injection loop of the Rheodyne valve was replaced with a trace enrichment cartridge (TEC), which has been described previously [8]. The Auto-Analyser Sampler and the Minipuls were modifed so that they could be controlled by the Spectrum microcomputer.

The hydraulic connections are shown in figure 1. The Minipuls was used to aspirate the sample from the sample cup into the donor channel of the dialyser block. Dialysate in the recipient channel of the dialyser block was pumped through the TEC by the 303/5SC pump (TEC pump). The Rheodyne valve could switch the TEC between the dialysate or HPLC solvent flow.

The operations of the sampler, Minipuls, injection valve, and 303/5SC pump are controlled by a BASIC program in the Spectrum microcomputer via the Z80A interface. The electrical connections between these components are schematically shown in figure 1.

### *Operation of the sample preparation unit*

The operations required to prepare a single sample for injection are shown in the timing diagram (figure 2) and are as follows:

(1) The sampler probe is moved from the wash reservoir into the sample cup, the Minipuls energized, and sample is drawn into the donor channel of the dialyser block. Once the donor channel is filled, the sampler probe is returned to the wash reservoir and the Minipuls is switched off.

(2) With the sample held in the donor channel of the dialyser, the TEC pump is energized and dialysate is drawn from the recipient channel of the dialyser and passed through the TEC. The TEC retains the analytes in the dialysate which have diffused across the Cuprophan membrane. The TEC pump is then switched off.

(3) The TEC is introduced into the solvent stream of the HPLC by rotating the Rheodyne valve to the inject position and the retained analytes eluted from the TEC onto the analytical column.

(4) With the Rehodyne valve in the inject position, both the Minipuls and TEC pump are energized to purge the sample and dialysate from the dialyser block and associated tubing.

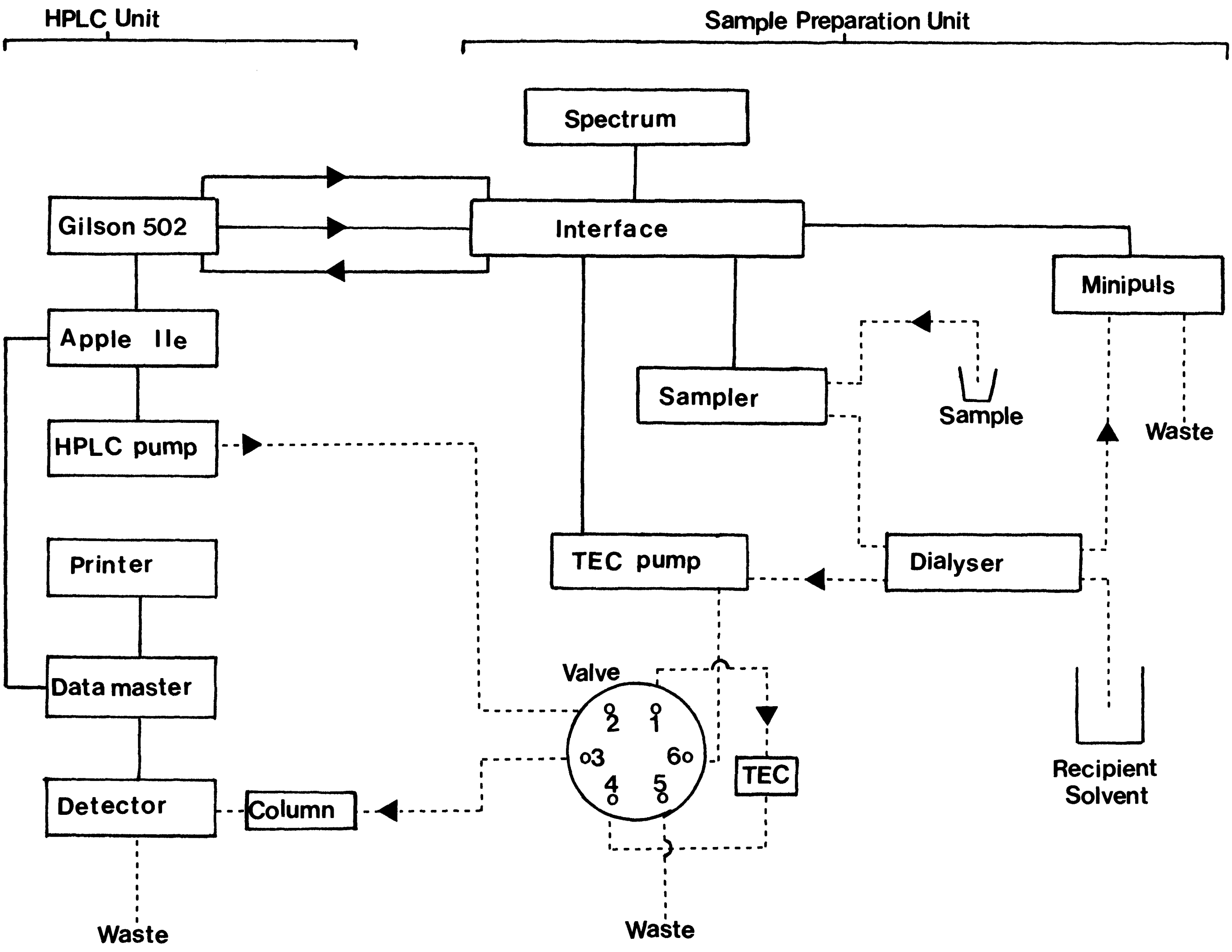

*Figure 1. Schematic diagram of the hydraulic (. . . . . .) and electrical (——) connections of the sample preparation and HPLC units.*

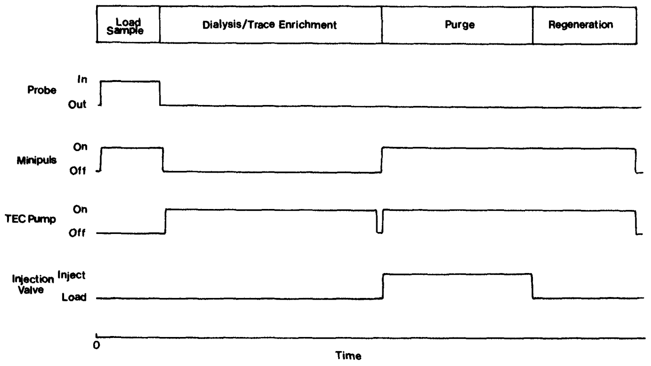

*Figure 2. Timing diagram for the operation of a single ASTED cycle to prepare a single sample*

(5) The Rheodyne valve is turned to the load position and recipient solvent passed through the TEC by the TEC pump. Both the Minipuls and TEC pumps are then switched off. In this way the retaining conditions on the TEC are regenerated by re-equilibrating with recipient solvent in preparation for the next sample.

*Configuration of the system*

The system consisted of the HPLC and the sample preparation units combined together (figure 1), so that the preparation and analysis of adjacent samples may operate concurrently. The flow diagram for this operation is shown in figure 3 and is shown schematically in figure 4. Once the first sample has been prepared and injected the processes only interact at the moment of injection, such that the faster process will wait for the slower. This allows the sample preparation unit to treat the next sample whilst the current sample is being chromatographed. The interactions between the two units during the injection sequence ensure that no samples are lost if either unit fails. Neither of the controlling programs needs to refer to the cycle time of the other process sequence and the Apple IIe controls the number of samples analysed.

The time taken for dialysis and trace enrichment depends on the amount of analyte required for HPLC analysis, the flow rate of recipient solvent, and the retention volume of the analyte on the TEC under the conditions used. Although efficient removal of analyte from the sample is achieved using long dialysis times and fast flow rates of

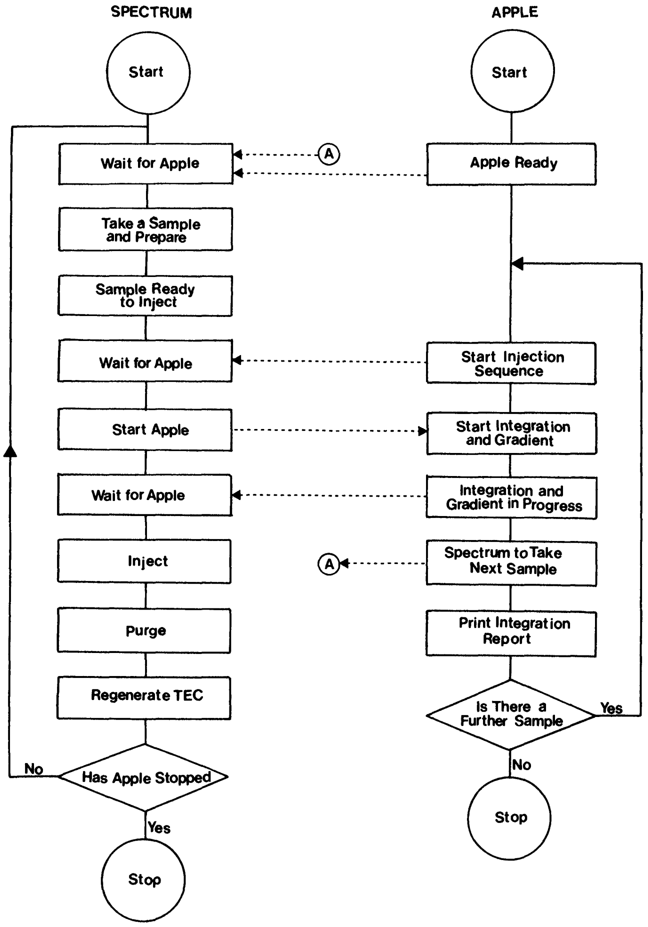

*Figure 3. Flow diagram of the algorithm to control the concurrent ASTED system. The Spectrum microcomputer controls the ASTED system and the Apple microcomputer controls the HPLC.*

recipient solvent, if the retention volume is exceeded retained analyte on the TEC will be gradually eluted and lost. Also the time taken for sample preparation, purging and regeneration should, ideally, be less than the time required to chromatograph the analyte. To optimize the system, the constant parameters, analyte retention volume and analytical sensitivity required, should be determined first, followed by the variables recipient solvent flow rate and dialysis time.

### Discussion

ASTED utilizes two separation methods, dialysis and trace enrichment, to selectively isolate analytes in a form suitable for subsequent HPLC analysis. Although classical dialysis is an efficient deproteinization technique, when applied to HPLC sample preparation it has the following limitations: (1) dialysis rate is proportional to the concentration gradient across the membrane; (2) at equilibrium the concentrations of the diffusable analytes across the membrane are equal; (3) the time taken to reach equilibrium may be up to 10 h. Thus usual dialysis techniques can provide either a high yield of solute in a large volume of solvent or a low yield of solute in a small volume of solvent. Serum dialysates have concentrations of analytes that are usually not detectable by HPLC analysis. When applying this technique to the sequential preparation of samples for HPLC, short dialysis times must be used which further reduces the yield of analytes.

The technique of ASTED overcomes this problem in two ways. Firstly, dialysis is performed by holding the sample stationary while the recipient solvent is moved continuously. This maintains a high concentration gradient

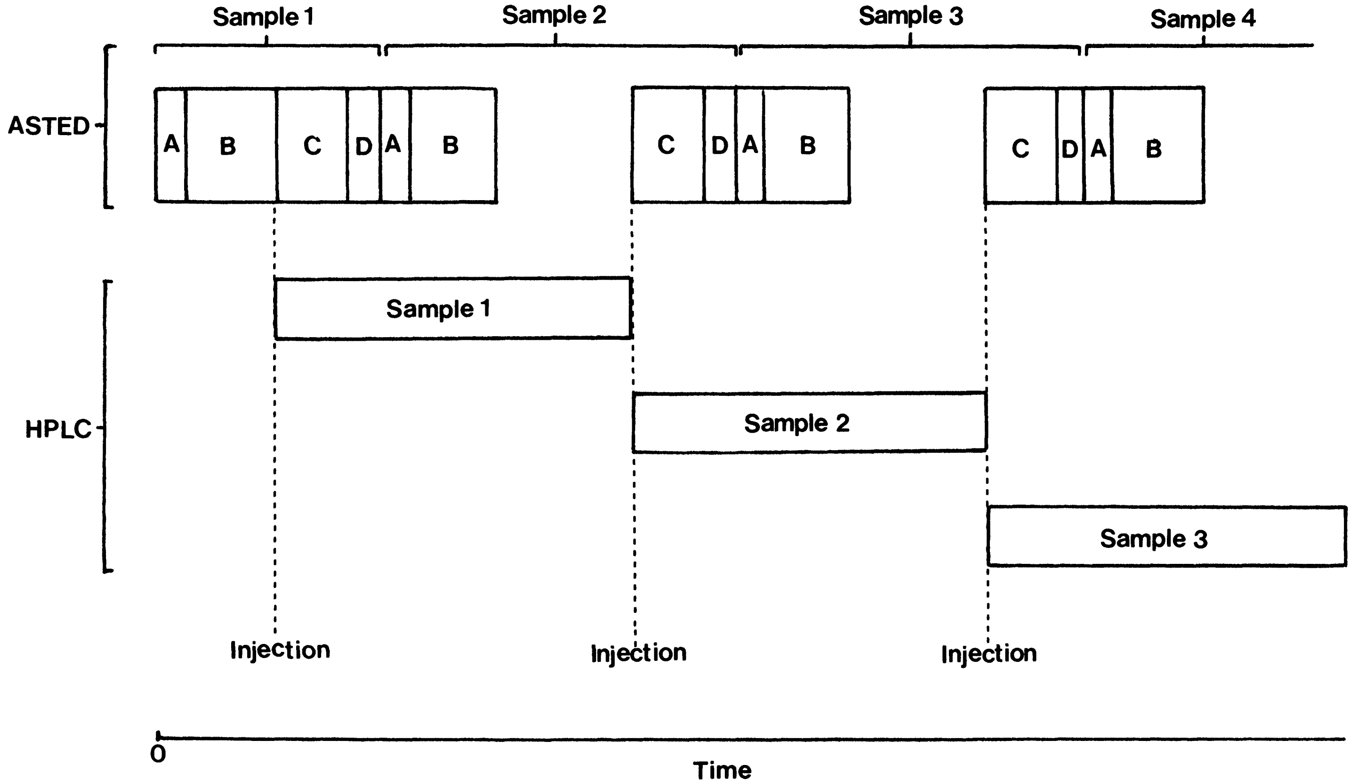

*Figure 4. Schematic diagram of the concurrent operation of the ASTED and HPLC systems. A = load sample; B = dialysis and trace enrichment; C = purge; D = regeneration of the TEC.*

*Table 1. Comparison of some features of ASTED with those of a typical batch sample preparation technique.*

| | ASTED | Batch |
|---|---|---|
| Volatile solvents required | No | Yes |
| Guard column required | No | Yes |
| Internal standard required | No | Yes |
| Can be regenerated | Yes | No |
| Running costs | Low | High |
| Flexibility of work schedule | High | Low |
| Efficiency of analytical control | Good | Poor |
| Staff time required | Low | High |

across the Cuprophan membrane, which results in a higher analyte yield in a shorter time than is possible when both donor and recipient streams are held static [10]. Secondly, the dilute analytes in the dialysate are retained on the trace enrichment cartridge so that they are available in a concentrated form for the HPLC injection. Due to the small physical size of the enrichment cartridge analytes have a very low retention volume in the analytical solvent and therefore are eluted in a small volume of solvent even when the TEC and analytical column are packed with the same material.

When compared with batch procedures for pre-treating samples, ASTED has many advantages (table 1). Moreover, when ASTED is operated concurrently with HPLC, it offers the added benefits of optimal use of equipment with rapid analysis of samples. With the exception of the first specimen, sample preparation occurs during the chromatographic separation of the previous sample (figure 4). Since the system is controlled according to the algorithm shown in figure 3, the operator does not have to consider the co-ordination of both processes. By the time the third sample is taken the system will have optimized its operation such that the speed of analysis is limited by the slower of the two processes (figure 4). Thus it is easy to interchange various ASTED and HPLC procedures of different lengths.

The use of sequential overlapping processes for sample preparation and HPLC theoretically provide higher analytical rates with less operator involvement than comparable batch preparation methods [11]. Although the individual steps of a batch process are simple to link together the resulting analytical system is difficult to control efficiently. If there is a failure in part of the sample preparation process, it is usually not detected until the HPLC results are obtained. At this stage much time and many samples would have been lost. In contrast, a failure within a sequential sample preparation process is detected almost immediately allowing appropriate action to be taken quickly with the loss of only the sample being treated at that time. This also permits a more flexible work schedule enabling rapid analysis of urgent specimens or repeat analyses.

At present there is no sample preparation method that achieves both total protection of the analytical column performance and elimination of interfering chromatographic components. Elimination of high molecular weight components using dialysis maintains the performance of the analytical column whilst trace enrichment presents the diffusible analytes in a form suitable for HPLC analysis. ASTED provides an effective sample preparation method for HPLC that is rapid, inexpensive and simple to operate.

### Acknowledgements

We wish to thank Gilson International, Villiers-Le-Bel, France for the loan of the HPLC apparatus and Anachem Ltd, Luton, Bedfordshire, UK for its maintenance.

### References

1. Adams, R. F., Schmidt, G. J. and Vandemark, F. L., *Journal of Chromatography*, **145** (1978), 275.

2. Turnell, D. C., Trevor, S. C. and Cooper, J. D. H., *Annals of Clinical Biochemistry*, **20** (1983), 37.

3. Blanchard, J., *Journal of Chromatography*, **226** (1981), 455.

4. Green, D. J. and Perlman, R. L., *Clinical Chemistry*, **26** (1980), 796.

5. Nordmeyer, F. R. and Hansen, L. D., *Analytical Chemistry*, **54** (1982), 2605.

6. Narasimhachari, N., *Journal of Chromatography*, **225** (1981), 189.

7. Apffel, A., Alfredson, T. V. and Majors, R. E., *Journal of Chromatography*, **206** (1981), 43.

8. Cooper, J. D. H. and Turnell, D. C., *Journal of Automatic Chemistry*, **7** (1985), 181.

9. Cooper, J. D. H. and Turnell, D. C., *Journal of Chromatography*, in press.

10. Turnell, D. C. and Cooper, J. D. H., *Journal of Automatic Chemistry*, **7** (1985), 177.

11. Zenie, F. H., *International Laboratory*, March (1985), 50.

